# A high throughput method for isolation of plasmatocytes from hemolymph of adult ***Drosophila***

**DOI:** 10.1371/journal.pone.0322707

**Published:** 2025-05-14

**Authors:** Mansi Yadav, Jassika Gupta, Debalina Chatterjee, Parul Mittal, Anju Shrivastava, Namita Agrawal

**Affiliations:** Department of Zoology, University of Delhi, New Delhi, India; BSRC Alexander Fleming: Biomedical Sciences Research Center Alexander Fleming, GREECE

## Abstract

Macrophages are essential innate immune system components, exhibiting diverse functions crucial for disease prevention. While much is known about their role in innate immunity, studying their functional versatility remains a challenge. *Drosophila* serves as an excellent model for investigating innate immune signaling due to parallels with vertebrate macrophages. Despite extensive research on larval plasmatocytes, there is a lack of sufficient published literature on adult plasmatocytes, hindering understanding of their contribution to late-onset diseases and aging. Current hemolymph extraction protocols from adult flies have limitations, making them less suitable for immunology experiments that require consistency and minimal stress on flies. Most established methods involve specialized tools such as nanoinjectors, silica capillary probes, etc. which are arduous and require advanced equipment. Although these approaches overcome some challenges in immune cell research, they remain complex and technically intricate. Here, for the first time, we present a protocol for quick, easy, cost-effective, and systematic isolation of adult *Drosophila* plasmatocytes by which we achieve nearly 2500 cells. We strongly suggest that this protocol might facilitate studies aiming to elucidate molecular and genetic mechanisms associated with immune response in various ailments enabling the exploration of immune cell dynamics, cell signaling and their role in disease progression.

## Introduction

Macrophages are an indispensable member of the innate immune system and are considered the first guard in the prevention of any minor to a major ailment [1]. From resident to circulating, from pro-inflammatory to anti-inflammatory and from promoting cell growth to inhibiting cell proliferation, there exist numerous versions of macrophages. Exploring and understanding the depth of functional diversity of macrophages remains one of the hotspots in science. Circulatory macrophages, confirmed by the markers present on their surface are considered mature cells, they encounter with the foreign particles and, thereby can be differentiated from the naïve macrophages that are present in the nurturing sites. The majority of life-threatening maladies spread their pathogenicity by affecting the innate immune arm first and therefore, the innate immune system serves as the primary component that responds to any danger posed by the diseases [2].

Interestingly, a remarkable parallel exists between *Drosophila* and vertebrate macrophages in terms of development and function and therefore, due to its highly tractable genetic system, *Drosophila* has been proven to be an excellent model to conduct studies of the inborn immune system and aging from more than three decades. The strength of *Drosophila* as a model system lies in its low maintenance cost, high reproductive rate, and short generation time of about 10 days at 25°C as well as short lifespan of around 90 days making it a desirable model for aging studies.

Hemocytes in *Drosophila* are made up of three types of cells: plasmatocytes, crystal cells and lamellocytes. Plasmatocytes are macrophage-like cells that secrete signaling peptides and extracellular matrix proteins in addition to phagocytosis (approximately 90–95% of total hemocytes). Crystal cells known for wound healing and melanization constitute the remaining pool of hemolymph. Lamellocytes, rarely found in healthy larvae, transdifferentiate in large numbers from plasmatocytes to encapsulate large pathogens [3,4].

Major research associated with plasmatocytes in *Drosophila* has been done in larval circulation, lymph gland and adult hematopoietic pockets [5,6]. However, not much of the literature exists on in vitro study of plasmatocytes isolated from adult circulation, may be due to a lesser amount of hemolymph in fly as compared to larva which makes its extraction difficult [7]. To monitor the role of plasmatocytes on disease advancement or senescence, its isolation during the adult stage is a major requisite. Existing protocols for hemolymph extraction have their drawbacks which makes them less favorable, especially for immunology experiments where reliability, viability, and minimal strain on the flies are the utmost criteria. Most published studies on hemolymph extraction have utilized specialized instruments such as nanoinjectors with micromanipulators, needle pullers and glass capillaries [8]. Other methods include an apparatus designed on the basis of airflow and pressure [9] a hemolymph sampling probe made up of silica capillaries mounted on a positioner for functionality [10] and the centrifugation technique, which necessitates large sample size [11]. These published methods, in the context of immune cells are an attempt to address limitations in their isolation but the techniques are tedious, labor-intensive, time-consuming and at the same time require a set of sophisticated instruments. Therefore, to overcome the constraints of published methods, we have standardized a credible and cost-effective protocol that focuses on the key properties and functions of plasmatocytes such as their adherence and phagocytic activity. The method has been carefully optimized for the in-vitro analysis of plasmatocytes isolated from the circulating hemolymph of adult flies. This abridged protocol might overcome the restrictions related to the low yield of plasmatocyte isolation with most of the available techniques. However, a large amount is required to conduct experiments to unravel molecular and genetic mechanisms underlying key biological processes including various diseases and immunity.

## Materials and methods

### *Drosophila* stocks

Wild-type *Drosophila* *melanogaster* (Oregon-R strain), HmlΔGal4 (BL#30139) and UAS-RFP (BL#30556) flies were maintained at a constant temperature of 25 ± 1⁰ C with 65% humidity on standard corn & agar meal under 12hrs light/12hrs dark cycle. Virgin females of Hml-Gal4 were mated with UAS-RFP males resulting in Hml-Gal4 > UAS-RFP expressing flies. To account for the sex-related differences, all experiments were conducted using 5–7 days old female flies. Each experiment was performed in triplicate and repeated three times to avoid variations.

### Isolation of plasmatocytes from the hemolymph of adult flies

**Fig 1:**
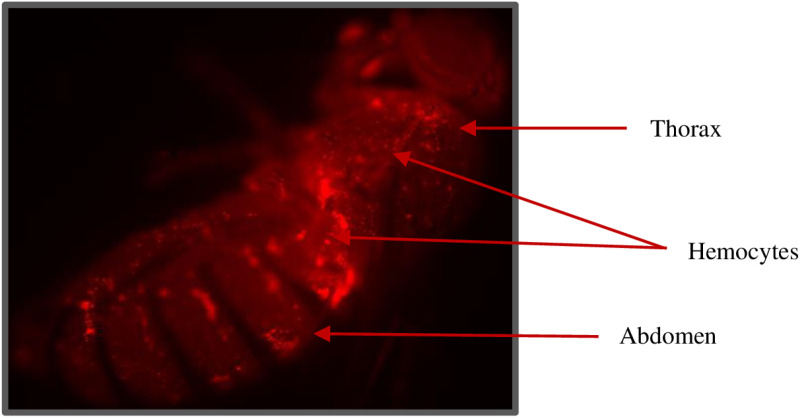
Hemocytes distribution in the adult fly. Fluorescence image of Hml-Gal4 > UAS-RFP flies showing the distribution of hemocytes in the thorax and abdomen. Magnification 10X.

To isolate plasmatocytes from hemolymph, Hml-Gal4 > UAS-RFP flies were observed under a fluorescence microscope to visualize the localization of hemocytes in different regions of flies (Fig 1). Based on the observation, we found that the high density of hemocytes is localized in the thorax and abdomen of adult flies and therefore, can be the best region to collect hemolymph.

**Fig 2:**
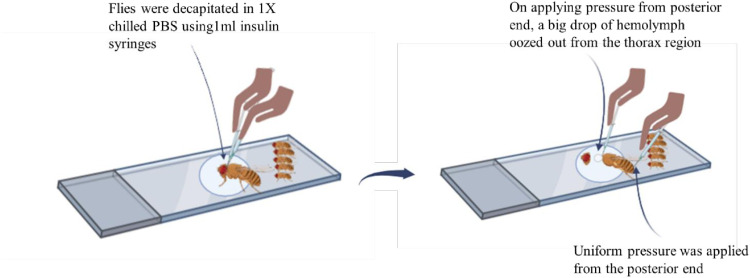
Illustration of the technique. Schematic representation of the protocol for plasmatocyte isolation from hemolymph of adult flies.

For hemolymph extraction and plasmatocytes isolation, female flies were selected to avoid sex-related differences and higher amount of hemolymph due to their larger size as compared to males which facilitates easier manual extraction [8]. Therefore, ten adult female flies were etherized, wings were removed with a pair of 1ml insulin syringes, and then vertically aligned in a row on a cavity slide. Each fly was individually decapitated in 60µl of chilled 1X PBS (phosphate buffered saline), followed by the application of uniform pressure from the posterior end of the abdomen using a 1ml insulin syringe so that the maximum amount of hemolymph could ooze out from the thorax. Additionally, to get the remaining plasmatocytes (Fig 1), thorax of the fly was punctured and then the flies were left to bleed for 10 seconds (Fig 2). The suspension was picked up by 20–200µl pipette and then plated on a pre-cleaned glass slide placed in a humidifying chamber to adhere for 1hour at 24⁰ C. Humidifying chamber is an enclosed space designed to maintain humidity to prevent desiccation of biological samples. In this protocol, we used a glass petri dish with moist Whatman paper and covered with another glass petri dish to maintain a relative humidity of 65–70%. Following incubation, the slide was then washed gently with 200µl of 1X PBS twice using a 20–200µl pipette to remove unadhered cells, fixed with 200µl of methanol for 30 seconds, and air dried until the methanol was completely evaporated. The slide was stained with 0.2% crystal violet for 90 seconds (for preparing 100ml of 0.2% crystal violet solution, 0.2g of crystal violet powder was weighed and added to a clean beaker. 50 ml of distilled water was initially added to dissolve crystal violet and the mixture was stirred until the dye was fully dissolved. Afterward, distilled water was further added to bring the total volume to 100ml. The solution was thoroughly mixed to ensure complete homogeneity and filtered using Whatman filter paper and stored in an air-tight amber bottle or covered with aluminium foil to protect from light), washed once with 200µl of 1X PBS to remove excess stain, and mounted with DPX/Vectashield mounting medium with DAPI.

### Identification of plasmatocytes

#### Using crystal violet staining.

Crystal violet is a positively charged dye that binds to proteins and DNA within a cell. It is a well-standardized and widely accepted stain to identify adhered plasmatocytes.

**Fig 3:**
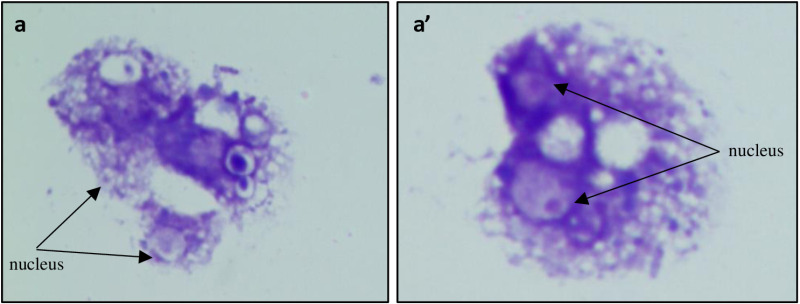
Plasmatocytes stained with crystal violet. Bright field images of plasmatocytes (a-a’) isolated from the hemolymph of adult fly, stained with crystal violet showing nucleus and cell membrane. Magnification 100X.

Isolated hemolymph was plated on a pre-cleaned slide placed in a humidifying chamber at 24⁰ C and adhered for 1 hour. It was then washed twice with 1X PBS to remove unadhered cells and fixed with methanol for 30 seconds followed by staining with 0.2% crystal violet stain for 90 seconds. The slide was then washed gently with 200µl of 1X PBS to remove excess stain, mounted with DPX and observed under Nikon bright field microscope (Fig 3 a-a’).

#### Phagocytosis assay.

Phagocytosis is an evolutionarily conserved mechanism that plays an important role in host defense and tissue homeostasis. Plasmatocytes which are the predominant hemocyte population and present in all developmental stages, display phagocytosis as one of their major functions [4]. Therefore, to further confirm if adhered cells and microscopically identified cells are indeed plasmatocytes, phagocytosis was performed. To assess phagocytosis, the following steps were conducted.

**Fig 4:**
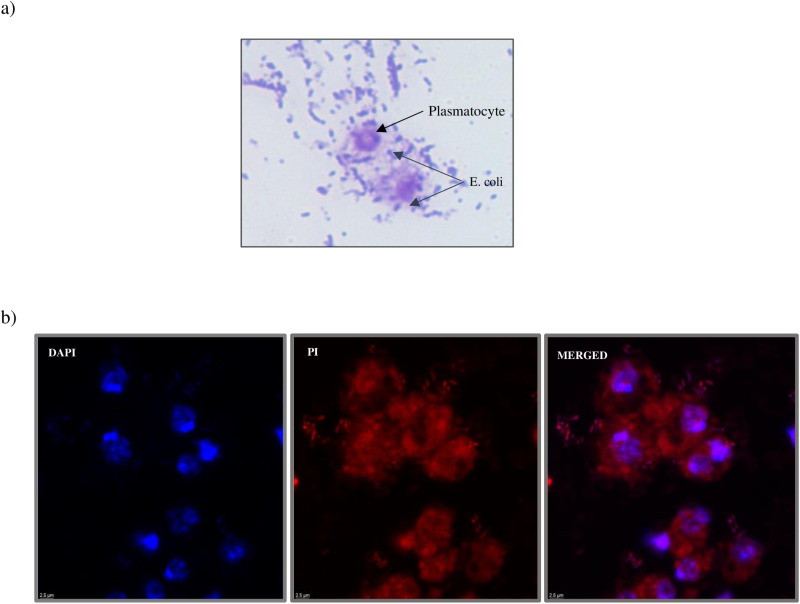
Phagocytic activity of plasmatocytes. Images of phagocytic activity of plasmatocytes a) Bright field image of plasmatocytes showing phagocytosis at 40X magnification b) Fluorescence images of plasmatocytes exhibiting phagocytosis at 100X magnification. Red = PI (Propidium Iodide) stain and Blue = DAPI. Please note that phagocytosis is specific to the cytoplasmic region of plasmatocytes.

i) Bacterial culture preparation: To monitor phagocytosis, primary culture of E. coli DH5α (E. coli strain DH5α) was selected due to its non-pathogenicity, well-characterized nature, and wide use in *Drosophila* research [12,13]. Bacterial culture was grown overnight in Luria broth (LB) media on an orbital shaker at 37⁰ C and 180 rpm. The following day, a secondary culture was prepared from the primary bacterial culture and absorbance was recorded at 600nm and culture was allowed to grow until it reached an O.D. of 1. The bacterial culture was centrifuged to remove excess LB and the pellet was washed thrice using 1X PBS at 8000 rpm for 10 minutes at room temperature. The bacteria cells were heat killed at 95⁰ C by incubating them in a dry bath for 1 hour and later resuspended in Schneider's media.
ii) Plasmatocytes were collected in 60µl of Schneider’s media to maintain the cell viability and adhered on a pre-cleaned slide as previously discussed. After washing unadhered cells, 350µl of bacterial culture was resuspended in 150µl of Schneider’s media to dilute the culture and was added to adhered plasmatocytes for 45 minutes. The slide was then washed gently with Schnieder’s media to remove excess bacterial cells. Plasmatocytes and bacterial cells were fixed with 200µl of methanol for 30 seconds and air dried. To observe under a bright field microscope, the slide was stained with 0.2% crystal violet and mounted with DPX (Fig 4a). However, to visualize phagocytosis under a fluorescence microscope, 10µg/ml Propidium Iodide (PI) dye was used to stain heat-killed bacterial cells for 2 hours at 37⁰ C on a shaker kept in the dark followed by centrifuging the bacterial cells at 11,000 rpm and washing it twice with Schneider’s media. The slide was then mounted with Vectashield mounting medium with DAPI (Fig 4b).

#### Enumeration of plasmatocyte viability.

Cell viability refers to the ability of cells to survive and maintain their normal functional and metabolic status under specific conditions. To assess cell viability, dye exclusion assay using trypan blue is widely used. Healthy cells possess intact cell membranes that prevent the trypan blue dye from crossing the membrane barrier whereas the dead or damaged cells with compromised membranes absorb the dye and appear blue under a bright field microscope.

To look into isolated plasmatocytes viability, dye exclusion assay using 0.4% trypan blue dye was performed. To prepare 0.4% trypan blue, 0.4g of trypan blue powder was measured and added to 100 ml of 1X PBS. The solution was stirred gently and filtered with a 0.2µm filter to remove contaminants.

**Fig 5:**
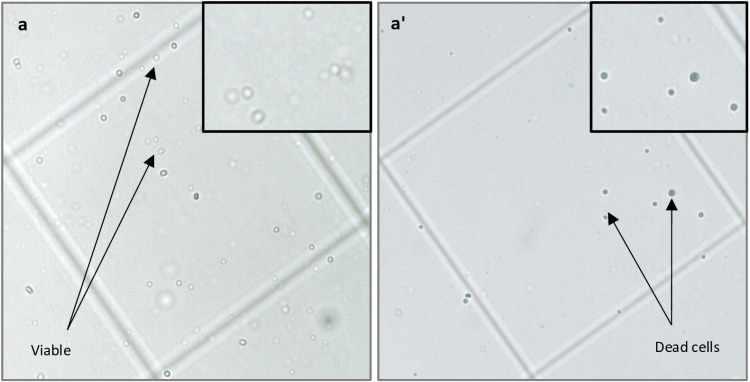
Viability of plasmatocytes. Bright field images of plasmatocytes on the hemocytometer grid (a) Immediately after loading the cells on the hemocytometer, all cells (100%) were found to be viable (a’) after 2 hours of incubation, 42% of the cells incorporated Trypan blue and appeared blue, indicating cell death. Representative images are captured using a 10X objective lens and zoomed to 0.76X, resulting in a total magnification of 7.6X.

To check cell viability, hemolymph was extracted in 1X PBS and cells were adhered in 96-well plate kept in a humidifying chamber for 1 hour incubation. Following incubation, the unadhered sample was decanted using a 20–200µl pipette, and 100µl of chilled 1X PBS was added and pipetted several times to mechanically detach the adhered cells. 10µl of this suspension was collected in a 500µl microcentrifuge tube and 10µl of 0.4% trypan blue was added. The microcentrifuge tube was placed on ice and after 2 minutes, 10µl of the mixture was loaded into a hemocytometer and observed under a bright field microscope (Fig 5a-a’).

#### Immunocytochemistry.

As a final validation for the identified plasmatocytes, plasmatocyte-specific P1 antibody (mouse monoclonal, gifted by I. Ando) was used. P1 antibody explicitly targets NimrodC1 (NimC1), a protein expressed specifically on the surface of matured plasmatocytes. NimC1 protein is a phagocytic receptor and therefore plays a significant role in the phagocytic ability of these cells [14].

**Fig 6:**
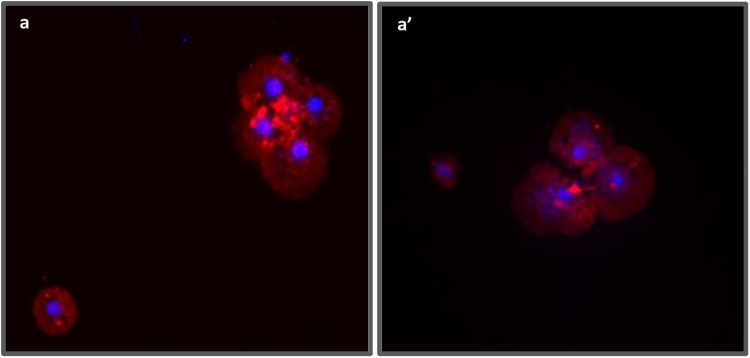
P1 antibody staining of plasmatocytes. Fluorescence images of different regions (a-a’) showing localization of NimC1 protein, a plasmatocyte-specific marker (P1antibody). Red = P1 protein and Blue = DAPI. Magnification 20X.

For immunocytochemistry, hemolymph isolated in 60µl of 1X PBS was plated on a sterilized round coverslip placed inside a 24-well plate and incubated for 1 hour. Cells were then washed with 200µl of 1X PBS and fixed with 200µl of methanol for 15 minutes on a shaker at room temperature. After fixation, methanol was decanted and the sample was air dried followed by blocking with 5% NGS (normal goat serum) in 0.2% PBST (0.2% Tween) solution for 2 hours on a shaker at room temperature. The blocking solution was decanted and cells were washed with 0.2% PBST thrice and then left for overnight incubation with P1 antibody (1:100 dilution in 0.1% PBST) on a shaker kept at 4⁰ C. After overnight incubation, the sample was washed thrice with 0.2% PBST and further incubated with Alexa fluor 546 (anti-mouse, at 1:250 dilution in 0.1% PBST) for 2 hours at 4⁰ C. The sample was again washed twice with 0.2% PBST to remove excess unbound secondary antibody. The coverslip was carefully taken out and mounted with Vectashield mounting medium with DAPI on a pre-cleaned slide. The prepared slide was observed and images were captured using a Zeiss fluorescence microscope (Fig 6).

## Results and discussion

Plasmatocytes, the *Drosophila* hemocyte lineage equivalent to vertebrate macrophages, play a crucial role in maintaining immune functions like clearing infections, cellular debris, and apoptotic bodies via phagocytosis [15]. Understanding their behavior and functionality is particularly important in the context of aging, where immune performance declines – a phenomenon referred to as immuno-senescence [16].

In *Drosophila*, aging significantly impacts immune system activity, with evidence that age-related changes can result in dysregulated immune response. Specifically, older flies may exhibit aberrant hyperactivation of antimicrobial defense mechanisms, coupled with a diminished ability to effectively combat infections [17,18]. These immune alterations not only compromise the organism’s resilience to infections but also contribute to promoting aging process.

Moreover, such dysregulations may also play a role in neurodegenerative diseases, like Alzheimer’s and Parkinson’s disease where peripheral inflammation exacerbates progression [19]. Here, hyperactivation of immune cells, such as plasmatocytes, leads to sustained inflammation which, in turn, accelerates neurodegenerative damage [20].

Given these insights, understanding how plasmatocytes function in both healthy and aging individuals is critical. Activated plasmatocytes trigger key signaling mechanisms, such as the JAK/STAT pathway, which plays a central role in orchestrating immune response by redirecting energy from metabolic pathways to bolster immune system functions [21]. Dysregulated activation of JAK/STAT pathways in these contexts may further perpetuate inflammatory signaling, worsening disease outcomes.

In this report, we have introduced a simple yet highly effective method for isolating plasmatocytes from *Drosophila,* a model organism extensively used to study macrophage behavior. Our protocol offers significant advantages over existing methods, making it a valuable tool for research on immune response in normal and impaired conditions. One of the major benefits of the protocol is its ability to isolate approximately 2500 cells from only ten flies, i.e., yielding nearly 250 cells from a single female adult fly. This number is more than sufficient to conduct variety of experimental assays, such as cell count, phagocytic activity, ROS estimation and mitochondrial functional analysis. These parameters are essential for understanding the role of the innate immune system in aging and diseases [5,22].

This method employs basic techniques such as manual decapitation and thoracic puncture with a pair of sharp syringes, which eliminates the need for strenuous and costly equipment. Thereby, the described protocol can be both accessible and easy to implement in various laboratories in present scenario with limited funding.

To further ensure the accuracy and consistency of the submitted protocol, we have conducted several validation experiments. These included experiments for plasmatocytes adherence, viability, phagocytic activity, and immunostaining with P1 antibody. The results finally confirmed that the method reliably isolates and identifies plasmatocytes, ensuring the validity of subsequent experiments.

Furthermore, the reproducibility, high yield, cell viability and adaptability of the proposed method make it particularly useful for experiments where sample size is a limiting factor, such as in disease conditions. To summarize, this method offers an easy, economical and good sample size with authenticity of plasmatocyte isolation for conducting studies related to innate immune system.
